# Experiences of hospice staff beyond the frontlines during COVID-19: A qualitative secondary analysis study

**DOI:** 10.1177/26323524251327804

**Published:** 2025-04-12

**Authors:** Thanga Harini Sundaramoorthy, John I. MacArtney, Abi Eccles

**Affiliations:** 1 Warwick Medical School, University of Warwick, Coventry, UK; 2 Warwick Applied Health, University of Warwick, Coventry, UK; 3 Nuffield Department of Primary Care Health Sciences, University of Oxford, UK

**Keywords:** COVID-19, palliative care, hospice, pandemic, healthcare professionals, emotional impact, qualitative research

## Abstract

**Background:**

The COVID-19 pandemic presented different challenges and work pressures for hospice staff. Staff who continued to work during the emergency public health period had to redefine not only their norms at work, but also the norms of their home and personal lives. Research on hospice staff, and broadly healthcare staff, mainly explores their professional roles and responsibilities, often overlooking the personal experiences and challenges that they encountered outside of this, especially during the first 2 years of the pandemic where their work and commitment persevered amidst unprecedented circumstances.

**Objective:**

To explore the impacts of the COVID-19 pandemic on the home life of hospice staff and identify opportunities to support staff in the future.

**Design:**

Qualitative interpretive secondary analysis study.

**Method:**

Reflexive thematic analysis of qualitative in-depth interviews of hospice staff (*n* = 25), conducted across seven hospices, to explore the impact and implications of the pandemic on hospice care.

**Results:**

Three main themes were identified: (1) Blurred margins: Participants discussed facing difficulty separating work and home life and taking stresses and thoughts about work home. (2) Isolation, loneliness and social interactions: Staff described being anxious and lonely during this period, and not feeling like socialising after demanding workdays. (3) Disruption to family and personal commitments: Some staff felt unable to see or support their parents, partners and children during the pandemic subsequently impacting their psychological and emotional well-being.

**Conclusion:**

Hospice staff compromised aspects of their personal life and family responsibilities during the pandemic to carry out their role at work. Workplaces and organisations should aim to support hospice staff more broadly to help with managing work-related pressures and balancing personal commitments in future emergency periods.

## Background

During the early stages of the COVID-19 pandemic in the United Kingdom, when lockdown and other emergency public health guidance were first introduced, providing specialist care became especially challenging for hospices and hospice staff.^1–3^ It is long-established that working in the hospice and palliative environment involves dealing with many stressors.^4^ Hospice staff are constantly exposed to others’ emotional and physical suffering, as well as dying and bereavement, thus managing one’s own emotions is a critical part of their role.^5–7^ However, the COVID-19 pandemic introduced different work pressures for hospice and healthcare professionals, and there has since been growing research interest into the emotional and psychosocial impact endured by hospice and palliative care staff during the pandemic. Although there is evidence that some staff thrived in the early period of increased camaraderie and “Dunkirk spirit,”^8^ most studies have focused on staff experiences of moral distress, emotional trauma and burnout.^9–12^ These studies primarily focus on the impact of changes to the responsibilities of hospice staff at work during the pandemic, and there has been little attention to the impact on their home life and the precautions that staff took to protect themselves and their family, friends and their patients.

Hospice care aims to improve the quality of life in individuals diagnosed with a life-limiting or terminal illness.^13^ In the United Kingdom, specialist palliative care is delivered in limited locations (such as hospitals, hospices, care homes and patients’ own homes) to manage complex palliative care problems that cannot be dealt with by generalist services. It includes providing timely relief from pain and distressing symptoms, offering a holistic support system to help patients and their families through illness and death, and access to specialist in-patient facilities.^14^ However, the advent of the COVID-19 pandemic posed an “existential crisis” to the core ideals of hospice care, as the virus threatened the amount and quality of life of patients, as well as affecting how staff sought to provide timely and holistic care.^8^

The COVID-19 pandemic also brought about uncertainty and disruptions in many aspects of individuals’ lives, daily routines, social activities, health of family and close ones in ways that meant individuals encountered a redefined understanding and experience of home during this time.^15^ Thus, during the lockdown periods, healthcare staff not only redefined their norms at work,^8^ but also at home. In recent decades, the concept of home has received increasing interest in exploring and understanding its multifaceted and dynamic nature to individuals.^16^ Commonly, individuals associate this term with their house or a structural place, their relationship with a person or object. However, Boccagni and Kusenbach discuss how individuals construct their perception of home across spatial, interpersonal and cultural relationships and practices.^17^ That is, what individuals call home represents a place of their normality, feeling a sense of belonging and associating it with a sense of security, familiarity and control over certain aspects of their lives.

Research has also demonstrated that individuals experience a sense of protection and security when they maintain social contact with family and friends and have social support to help protect against loneliness.^18,19^ Indeed, studies have shown increased loneliness as a result of the pandemic in the general population and among healthcare workers.^20,21^ Here, we discuss loneliness as commonly described by the feeling when a person’s social needs are unmet by the quantity or quality of their social relationships.^22,23^ This is different to being alone where one is not around other people, which was also noted during the pandemic as some staff changed their living arrangements to live alone.^21^

This qualitative secondary analysis aims to supplement existing research into the impacts of the COVID-19 pandemic on hospice staff by examining the effects on the home and personal lives of hospice staff. Exploring these aspects will enhance our understanding of the effects of the pandemic on the roles, experiences and emotional challenges encountered by frontline staff. In doing so, we will identify ways that hospice – and healthcare staff in general – might be better supported in future pandemic emergencies, and beyond.

## Methods

### Design

A qualitative interpretive secondary analysis of interviews with hospice staff collected during the primary study entitled the “Impact and implications of COVID-19 on the relational, social and healthcare experiences of Hospice care” (ICoH) study.^24^

### Primary study setting, data collection and sample characteristics

During the ICoH study patients, carers, staff and senior managers were purposively sampled from hospices in the West Midlands, UK. Seven hospices successfully recruited staff participants and a maximum variation approach was used to select participants for interview. This purposive sampling and maximum variation approach, commonly used in qualitative research, involves identifying key dimensions of variability and intentionally selecting participants to increase the heterogeneity of the experiences collected.^25^ In the primary study, participants were intentionally selected to include individuals across a range of roles in the hospice including clinical, administrative and support positions, and individuals across different age groups and genders.

Key participant characteristics are summarised in Table 1. Included in the participant group are doctors (including speciality trainees and consultants), nurses (including support and care assistants), allied health professionals (including social care workers and psychological, occupational, physio and complementary therapists) and support services staff (including administrative and volunteer coordinators).

**Table 1.** Participant characteristics.

Category	Subcategory	*N*	%
Gender	Female	23	92
	Male	2	8
Ethnicity	White-British	25	100
Age	18–29	0	0
	30–39	7	28
	40–49	4	16
	50–59	13	52
	60–69	1	4
Role	Doctors	2	7
	Nurses	12	48
	Allied health professionals	7	28
	Support services	4	16

Source: Adapted from ICoH study staff report by MacArtney et al.^9^

Audio recorded informed verbal consent was provided by participants and in-depth qualitative interviews with participants were conducted by JM, AE and other members of the primary ICoH study team between August 2021 and February 2022. We used audio recorded consent to make remote participation as accessible and convenient as possible in a context where participants were very busy and adhering to social distancing restrictions.

The interviews followed an in-depth approach because little was known about the lived experiences of those providing hospice care during the emergency public health period of the pandemic. All interviews started with an open question, “Could you tell me a bit about your background and what kind of contact you have with the hospice during the pandemic?” Follow-up questions were in response to the participant’s story which would include prompts on accessing services, experiences of different locations of care, concerns about COVID-19 or the impact of the pandemic on care and family. Analysis of the primary data highlighted significant impacts on staff’s personal lives, prompting this secondary analysis study to re-examine the interviews in relation to their home life.

Interviews were conducted via videocall or telephone and were recorded and transcribed. All data were pseudonymised and held by the chief investigator of the ICoH study (JM). Hospice staff interview transcripts (*n* = 25) were internally shared with the lead author (THS) for use in the secondary analysis.

### Secondary data analysis

Analysis of the interviews in this study allowed us to revisit the real-time documented experiences and challenges that staff faced at home during the unprecedented circumstances of the emergency public health period of the COVID-19 pandemic to better understand the impact on the home and personal lives of staff and how their experiences and perspectives can help inform policy and practice interventions that aim to support the well-being of staff.^26^

Interview transcripts were initially read and assessed by the lead author (THS) for quality of data, in terms of having sufficient reflection and discussion on how working during the pandemic impacted their personal lives. Some staff were furloughed, but this study focuses on those who continued to work during the pandemic as we are examining the impact of work on home life.

The initial reading of transcripts also provided the opportunity to become familiar with the dataset and create an initial set of codes for this study. Once familiar with the dataset, it was annotated, and reflexive thematic analysis was undertaken,^27^ using NVivo R1 (a qualitative data management computer software produced by Lumivero (formerly QSR International)). An abductive approach was taken to inform coding and analysis and capture idiosyncratic participants’ experiences and feelings.^28^ The analysis is reported following Braun and Clarke’s Reflexive Thematic Analysis Reporting Guidelines.^29^

Once transcripts were coded, a concept map was developed to scrutinise the excerpts and identify patterns.^30^ The ongoing development of codes was an iterative process, involving regular meetings with AE (supervisor) to discuss the development of the project, the codes and the themes. Maintaining a reflexive journal and comparing interpretations of the data with the co-authors, allowed sharing and critiquing of different perceptions and insights. THS led the writing-up of the findings, with all authors contributing to the analysis and paper’s development.

### Ethical approval

Ethical approval was granted by the Biomedical and Scientific Research Ethics Committee (BSREC) of University of Warwick for the original study (BSREC 98/20–21), as well as for the secondary analysis (BSREC 138/22-23).

## Results

The pandemic impacted the day-to-day working lives of hospice staff, which has been reported in-depth in the original study.^8,9^ Expanding further, this study demonstrates how the staff members’ home lives were substantially affected in ways that have not yet been documented. Three main themes related to “impacts on home life” were identified during analysis: (a) blurred margins, (b) isolation, loneliness and social interactions and (c) disruption to family and personal commitments.

### Blurred margins

Healthcare professionals prioritised setting boundaries between their professional and personal lives as part of their core principles. However, the COVID-19 pandemic made maintaining these boundaries particularly difficult. One participant shared this feeling directly:

. . . I think it’s embedded into us as nurses that you know you don’t let your life at work impact home and vice versa, but it was just more difficult during the pandemic. (ICOH47, senior nurse)

Specifically, participants working from home and consulting with patients virtually during the COVID-19 pandemic discussed the loss of a physical boundary. They shared how different and “strange” their home atmosphere became during this period (ICOH56, allied health professional), and this was often attributed to a lack of physical separation between work activities and the home environment:

You know you would be sat at the end of a phone at the end of your dining table so it did feel strange. (ICOH56, allied health professional)

There were times when I look over at the sofa and I’d think, how are we gonna manage this tonight?. . . . So it was like not having that space where you can go away and kind of do that. . . . getting used to that essence being in the room at the end of the day and not having that separation. (ICOH30, senior nurse)

The space where staff usually go to physically and emotionally unwind after work has been interfered upon by work activities, making it more difficult to separate between work and personal responsibilities.

Additionally, staff members who worked remotely, particularly staff who lived alone, mutually shared the feeling of holding onto these thoughts and emotions without being able to reflect with colleagues as they would do before the pandemic when working in the same physical space:

I live on my own so I think often when you’re having these really deep emotional conversations with people, you need to kind of get together as a group and debrief and just kind of be with someone. . . . and that’s all taken away from you then. You are kind of sat with all these emotions that you’re holding for [the] person and then in 10 minutes time you’ve got another phone call again. So it was, it was really difficult. (ICOH56, allied health professional)

. . . it’s [working from home] made me feel really isolated. . . . I’ve never worked from home before and individually, I’m quite a, I like to be around people and I like working in an office and that’s how I get my kind of I suppose my energy and my motivation to do stuff and I just find it really alien to then just be sitting at a desk on my own. (ICOH68, allied health professional)

I think you could feel like you’re trying to juggle . . . and hold and contain all of these emotions sitting in your living room . . . (ICOH30, senior nurse)

Staff working on site at hospices were also not able to debrief with their work colleagues in the same way as before the pandemic due to physical distancing and infection control measures within the hospice setting. This meant they were taking reflections on patient encounters home:

There’s a lot of support that goes on in those lunch breaks, and those, you know, afternoon coffees where it might just be a little bit quieter and they . . . probably don’t even realise that they’re reflecting, but that just didn’t that that didn’t happen because people weren’t coming back. They were taking it home with them, which was hard. (ICOH62, senior nurse)

One participant, in particular, who had started a new role shared the main factors affecting her at the time and becoming tearful after work:

. . . but I felt that I had two things to get to grips with. First of all was COVID and then secondly was this new role of, you know, being expected to be in charge of a section of the ward, I meant not to begin with. Obviously they let me find my feet, but. Yeah, that was really quite scary and then I admit to having quite a few tearful journeys home feeling just useless. (ICOH 45, nurse)

Some working at hospices and with patients in the community during the pandemic shared that the changes to practices meant they could not easily follow-up or support patients in the same way:

You come home with this sense of you’re dealing with what you know about and what you can do, but you just have this unease about what you know is there, but you don’t know about. (ICOH48, doctor)

And personally, I actually really struggled with that, because, I knew that in the context of the whole country, that lots of people were dying and here was I with lots of skills and looking after people who are dying and you know not using them as much as I might have been able to do in a different setting. (ICOH57, doctor)

The emotional impact of carrying work-related thoughts and feelings home for staff was profound. Without being able to discuss patient encounters in the same way they used to, staff felt unease and were often having to manage their emotions by themselves which came with implications of its own, as will be outlined in the next section.

### Isolation, loneliness and social interactions

Staff who were physically working with patients in the community and in the hospice discussed how they adjusted their living arrangements to live in a separate household to their partners and children or stay isolated from family members in the same household to keep everyone safe and avoid passing infection. Participants described being “very fearful for the first few months” (ICOH67, allied health professional) and “a sense of permanent state of being on alert, battle ready” (ICOH61, nurse) with regards to infection control. Staff purposefully keeping social interactions to a minimum was often attributed to these worries:

. . . I stay with my mum and who is sort of in her late 70s so there was a big worry for me about taking something home to her too. So I ended up sort of isolating in my room and not going into the rest of the house. (ICOH34, nurse)

I was too scared to visit them [participant’s parents] in case I brought something in from the hospice to them. They were both shielding because mum has breast cancer, and yeah, it was difficult. (ICOH50, allied health professional)

Staff were, therefore, often cautious and protective of their loved ones and prioritised keeping them safe during the pandemic, even if this meant limited social contact as a consequence.

During this difficult time, staff were also dealing with social isolation and loneliness at home. Several participants shared missing the presence of and having downtime with family members after demanding work shifts and managing their personal struggles, including anxiety. One participant shared:

So actually when I got home after a shift, I felt very lonely. We’d have a busy shift, I was overwhelmed and suffering with anxiety, and then often I would come back to an empty house and that was hard. It was a difficult time, it really was. (ICOH50, allied health professional)

Another participant shared how the precautions taken meant they had not seen their spouse in almost a year and their daughter lived in a separate household (ICOH34, nurse). They also talked about how they missed the comfort of human touch and the toll this took on them:

. . . that was a real sort of kind of sit up and realise, oh my God I’ve not actually touched somebody with my own hands for months and that and that was real, that was really hard, really hard. . . . You keep going. You keep going. You keep going and you don’t realise and then and then all of a sudden it’s like well. Gosh, you know I’ve got this far down the line and you know have a chat with myself. You know what? What are you doing? What’s going on? You can’t carry on, that’s not sustainable, you know? (ICOH34, nurse)

An important role of healthcare staff is to provide personal care and support; however, not being able to have any human touch or physical contact at work or at home due to infection control measures was particularly difficult for many staff members and seems to be unsustainable as time passes.

Participants also shared how working through the pandemic impacted their social lives in various ways. Some lacked motivation to partake in social interactions, even when presented with opportunities to do so. One participant attributed this to dealing with sensitive situations with patients and their families at work, so they did not have the motivation to socialise after work as well:

You know, I’ve come in this morning and I’ve already dealt with a safeguarding and a deprivation of liberty. You know, I didn’t know at six o’clock this morning that’s what I’d be dealing with. That’s tiring enough. Working with people is tiring enough. You know, I sometimes get home and think I don’t actually want to talk to anybody else today [be]cause that’s literally all I’ve done all day is talk . . . (ICOH67, allied health professional)

Another participant shared that a combination of factors from the tiredness of working through the pandemic and concerns about passing infection to people at home contributed to the feeling of not wanting to meet with people and socialise:

Moving forward when the restrictions were lifted a bit, you know, but it was that tiredness I suppose starts to sort of creep in. Life was trying to return to normal, you know you worry more about mixing with people at home because of where you work, and so you know people would want to meet up. They would want to and I’m like, yeah, I don’t think I do. You know, I, I don’t think I want to do that. And you know, there’s that worry that you kind of come across a little bit antisocial. (ICOH62, manager)

The diminished motivation and concerns of hospice staff regarding social interactions further compounded the emotional and mental strain they were experiencing at work and in their personal lives.

### Disruption to family and personal commitments

When the government announced the first lockdown, several participants found changes to their working circumstances challenging to adapt to. Participants discussed difficulties in changes to their work commitments and how this impacted their daily activities. One participant, working from home, discussed the challenge of managing between childcare and remote working commitments:

You’d be in an important meeting and he [participant’s child] would just be like crying in the background and I felt guilty as a parent [be]cause I thought gosh, they’re just being neglected for hours on end, when this is a time when they should be getting really key stimulation that they need and we’re just not even talking to them for 2-3 hours because we’re both on meetings back to back. And it was a time when we felt like we had to justify ourselves in a work sense more than ever, because there was that that fear of redundancy looming over your head. (ICOH68, allied health professional)

One participant stated that she initially struggled to arrange care for her newborn daughter; however, her workplace had helped to arrange a schedule so she could split caring responsibilities between herself and her partner:

And so I actually came back from maternity leave . . . I did about three weeks and then ended up being in lockdown and so my little girl I was taking her to my mum’s. My mum and dad in the 70s. They ended up shielding so I couldn’t take her there and so I basically asked, I only do two days, two long days. I said can I actually do Saturday, Sunday, Monday, two out of those three and then my partner could work Tuesday, Wednesday, Thursday, Friday and actually they were really good. They were really flexible . . . (ICOH60, nurse)

Managing personal commitments, alongside changes to work commitments, was challenging for many participants. Among amending the management of family commitments, organising childcare for staff who are parents was particularly difficult. As part of the government’s actions to support healthcare workers’ families from March 2020, the time of the initial lockdown, staff members were able to send their children to nursery and school. As mentioned above, one participant had support from her workplace whereby she was able to work certain days of the week and her partner the remaining days of the week so they could split childcare during the week (ICOH60, nurse). The participant shared that eventually she was able to send her child to nursery; however, it was tough having to manage attending work and leaving their child at home without parental supervision before this:

. . . and eventually I put her into nursery, which is fine, but yeah, it was really tough [be]cause I don’t know what to do. (ICOH60, nurse)

As infection control measures were eased during the pandemic, hybrid working was introduced where staff could work with their colleagues in person on some days and work remotely on other days with time to spend with their families and on personal commitments. One participant shared:

. . . it works quite well having the mix because it the days I’m working from home I’m able to take my son to school and still get back for nine o’clock to work from home. Whereas if I was going into hospice, I’d be late, you know, getting to school and then going in. So me and my partner can still share that a bit more. It’s a bit, so it does make sort of your home life balance a bit easier . . . (ICOH68, allied health professional)

These experiences demonstrate how participants managed to find a way to balance their family and personal commitments during the pandemic.

For some staff members, them and their family faced challenges regarding managing the losses of people close to them. Several hospice staff had family members who were clinically vulnerable to COVID-19, and some experienced personal bereavements, both related and not related with COVID-19, during the pandemic. Participants talked about how the difficulties they faced during this time were exacerbated due to their work commitments. Some discussed the distress of not being able to support their own family members who were critically unwell and dying because of work commitments and infection control measures, avoiding mixing between households:

. . . I was doing early and lates which was a massive shock. Obviously that changed things at home as well, because I care for my mum and dad. I go and visit them every morning and every night, but obviously that had to stop, because I was on an early shift or a late shift. (ICOH50, allied health professional)

. . . at the time my dad had cancer. . . . They [participant’s parents] were scared of having anybody in their house um and my mum slept on the floor for six months by my dad’s bed so. . . . Yeah, I’ve moved and house and job specifically so I could be on hand to care for them and that was taken away by covid and by the role that I did. (ICOH75, allied health professional)

Some participants talked about feeling helpless and upset during this situation and the psychological and emotional impact on them:

. . . And I think because this is the role that you train for. If you can’t deliver that to your own parents, that has a big effect on you psychologically (ICOH75, allied health professional)

I think the, personally for me it was difficult, as I’d experienced. My first grandchild, we lost our first grandchild during covid. I therefore couldn’t support my son and his wife through that because of not being able to have any contact. That emotionally did upset me, of course. I do feel that I couldn’t support my family members as well as what I would have normally been able to do. (ICOH47, nurse)

These experiences have altered the role and identity of individuals in their personal lives, as staff dealt with feelings of helplessness, leading to potentially long-lasting emotional and psychological impacts.

## Discussion

Hospice staff compromised aspects of their home life and family responsibilities during the COVID-19 pandemic emergency public health period to carry out their role at work, which on its own came with additional pressures. The findings of this study highlight the profound impacts the early pandemic had on the home lives of many hospice staff. Staff were coping with many emotions regarding various matters during the pandemic, including managing work on their own as opposed to alongside the team, being fearful of spreading infection to people at home, and not being able to support family with personal losses in the same way.

### Comparison of findings with previous work

An overarching theme of isolation (through physical separation from others) and the development of loneliness, incurred largely due to infection control measures, was recognised during analysis. Accordingly, a study in Australia identified that loneliness was common among clinical staff, including doctors, nurses and allied health workers, compared to other healthcare professionals (such as administration workers, researchers and cleaners).^21^ Furthermore, limited social contact, including with family, friends and partners, increased the odds of loneliness among these personnel^21^; meanwhile, social support from family and work support were protective and supportive factors for staff and reduced their stress levels.^31,32^ A study in London suggested that limiting interactions to five to six daily contacts per person (45%–55% reduction in pre-COVID-19 average daily contacts) can keep COVID-19 suppressed and avoid COVID-19 waves in the study’s healthcare system.^33^ Indeed, these findings must be considered in future pandemics and emergency periods, subject to the appropriate infective agent, to identify whether some and limited interactions may be feasible as protective factors for staff.

A recent study examined journal entries of healthcare staff and involved encounters and feelings regarding their own losses during the COVID-19 pandemic that included anger, sadness and guilt contributing to emotional distress, supporting the findings of this study.^34^ Staff shared the feeling of not being able to support family members at the end of their lives in the same way as the way they are trained to do in the workplace. Staff were often mourning their personal losses in isolation due to geographic separation from them. In our study, one participant mentioned that they had only realised how long it had been since they held someone’s hand until they held the hand of their deceased relative, which further added to the feeling of physical isolation and emotional distress. Moreover, Wang et al. show that losing a spouse to COVID-19 is associated with higher levels of self-reported depression and loneliness compared to pre-pandemic associations.^35^ Here, the complication of grieving and mourning due to social isolation, financial insecurity and lack of practical and emotional support, among other factors made the experience very distressing for individuals.^35^ It is, therefore, important that we understand the significance and the impact of bereavement and grief on the mental health and well-being of healthcare staff, especially during the context of a pandemic.

### Strengths and limitations

In-depth qualitative interview data allowed an analysis to identify challenges experienced across different parts of hospice staff members’ lives. The interviews were conducted when there were still some public health guidance in place or soon after more stringent restrictions were lifted, documenting raw and detailed participant experiences and insights from working during the pandemic.^2^ The recollections during the interviews discuss the transition into the first lockdown (March 2020) and the relaxation of public health protections (“freedom day”; in July 2021) and afterwards, although most hospices continued their measures throughout the data collection period.

During data collection in the ICoH study, participants were asked limited questions regarding emotional impacts as the ICoH study, and its interviews had broader aims to address compared to the current study. These, in addition to interview time limits, meant certain concepts (such as social relationships) that were only briefly discussed during the interviews could not be explored. However, the salience of conducting the interviews during the emergency public health period of the pandemic itself, rather than retrospectively, helped mitigate this limitation by providing an accurate and descriptive account of staff experiences that might have been less accurately or vividly shared in hindsight.

Although maximum variation sampling was sought during recruitment in the ICoH study, diversity in gender and ethnicity was limited. The researchers in the primary study did seek a more ethnically diverse sample from the hospices; however, the reasons why staff from these groups chose not to participate were not collected by the original study researchers. As participation was entirely voluntary, we were limited to those who were willing to take part in the study. Jesuthasan et al. reported the complex manner in which the well-being of healthcare workers of Black, Asian and other ethnic minority backgrounds was affected during the COVID-19 pandemic. While female healthcare workers formed the majority of the participant group here also, the authors discussed the staff’s emotional burden of racial inequality and cultural insensitivity.^36^ Therefore, research on the impact of future pandemics should aim to recruit a diverse participant group, including expertise, gender and ethnic background.

### Recommendations

Research is needed into the ongoing implications of working through the public health emergency period of the pandemic. The focus should be aimed at proactively supporting staff’s health and welfare,^37^ but this needs to explore the implications for staff mental and emotional well-being both at work and at home.^38^ This can include offering ways to debrief (such as Schwartz rounds or talking therapies after difficult patient encounters), childcare support for staff with young children, and opportunities for flexible and hybrid working. Furthermore, the long-term consequences of loss, loneliness, depression and other mental health effects from the pandemic are crucial to recognise and manage in the current post-public health emergency period.^39^

Recommendations to support staff through future emergency public health periods will need to consider the wider implications of their work on their personal and home life. This could include mandating longer periods for compassionate leave during times of national crisis, with a phased return to work and other flexible working options. To help preserve some social normalcy among individuals during a pandemic, more consideration could be given to balancing staff’s psychological need for social interaction with the effects of increased contacts upon transmission of the pathogen upon surges or waves of infection.

## Conclusion

This study highlighted how the pandemic impacted various aspects of hospice staff’s home and personal lives, including their home environments, social connections and personal responsibilities. Staff sacrificed many of their responsibilities to prioritise their commitment to work and infection control and managing substantial changes to both work and home duties during the COVID-19 pandemic was particularly challenging for them, often leaving them to cope with difficult emotions on their own causing emotional distress. Learning from these experiences, taking proactive measures to prevent the need for drastic infection control protocols and providing comprehensive welfare support for staff can help safeguard our hospice care workforce to look after themselves and vulnerable patients during future emergency periods.

## Statements and Declarations

**Ethical Considerations:** Ethical approval for the primary data collection study was provided by the Biomedical and Scientific Research Ethics Committee (BSREC) of University of Warwick (BSREC 98/20–21), as well as for the secondary analysis study (BSREC 138/22–23). Consent to participate was collected as part of the primary data collection study (BSREC 98/20–21).

**Consent for Publication:** Collected as part of primary data collection study (BSREC 98/20–21).
